# Cashew Nut Oil Improves Lipid Metabolism and Fat Liver Deposition in High‐Fat Diet‐Fed C57BL/6J Mice

**DOI:** 10.1002/lipd.70015

**Published:** 2025-10-17

**Authors:** Marcella Duarte Villas Mishima, Andressa Ladeira Bernardes, Carlos Alexandre Pinheiro, Luisa Gouveia Lana, Iasmim Xisto Campos, Valéria Silva de Lana, Izabela Maria Montezano de Carvalho, Patrícia Fontes Pinheiro, Renata Celi Lopes Toledo, Ana Paula Dionísio, Helen Hermana Hermsdorff, Maria do Carmo Gouveia Peluzio

**Affiliations:** ^1^ Laboratory of Nutritional Biochemistry, Department of Nutrition and Health Universidade Federal de Viçosa Viçosa Brazil; ^2^ Laboratory Food Analysis, Department of Nutrition and Health Universidade Federal de Viçosa Viçosa Brazil; ^3^ Department of Chemistry Universidade Federal de Viçosa Viçosa Brazil; ^4^ Laboratory of Experimental Nutrition, Department of Nutrition and Health Universidade Federal de Viçosa Viçosa Brazil; ^5^ Embrapa Agroindústria Tropical Fortaleza Brazil; ^6^ Laboratory of Clinical Analysis and Genomics, Department of Nutrition and Health Universidade Federal de Viçosa Viçosa Brazil; ^7^ Laboratory of Energy Metabolism and Body Composition, Department of Nutrition and Health Universidade Federal de Viçosa Viçosa Brazil

**Keywords:** *Annacardium occidentale* L., lipid profile, metabolic effects, oleic acid, unsaturated fatty acid, vegetable oil

## Abstract

Cashew nut oil adds value to the production chain of this Brazilian oilseed. It is a good source of monounsaturated and polyunsaturated fatty acids and bioactive compounds, such as phenolics. This study investigated the effects of cashew nut oil on the metabolism of animals subjected to a high‐fat diet. C57BL/6J mice were fed a high‐fat diet (12 weeks), then switched to diets containing cashew nut oil, soybean oil, or lard (12 weeks). Diets were characterized for their fatty acid profile. General parameters, metabolic markers, oxidative stress, gene expression, and liver steatosis were evaluated. Oleic acid was predominant in the cashew nut oil‐added diet, linoleic acid in the soybean oil‐added diet, and palmitic acid in the lard‐added diet. Cashew nut oil reduced blood glucose, triglycerides, uric acid, and liver steatosis, and increased SOD expression and activity and catalase activity. Soybean oil reduced blood glucose, total cholesterol, LDL cholesterol, triglycerides, and liver steatosis. Both vegetable oils, rich in unsaturated fatty acids, demonstrated more benefits than animal fat. Cashew nut oil effects may be mediated by the reduction of hepatic fat accumulation and oxidative stress, leading to lower triglyceride levels, improved insulin signaling, and decreased uric acid, probably due to its fatty acid composition, rich in oleic acid. Phenolic compounds may enhance its antioxidant capacity. The quality of fatty acids and the bioactive compounds is a determinant of the metabolic effect of oils. Cashew nut oil may improve lipid metabolism, reduce liver steatosis, and enhance antioxidant effects.

## Graphical abstract

Thirty C57BL/6J mice were fed a high‐fat diet for 12 weeks to induce metabolic changes. They were then switched to diets containing cashew nut oil, soybean oil, or lard for another 12 weeks. Soybean oil reduced blood glucose, total cholesterol, LDL cholesterol, triglycerides, and liver steatosis. Cashew nut oil reduced blood glucose, triglycerides, uric acid, and liver steatosis, and increased SOD expression and activity as well as catalase activity.

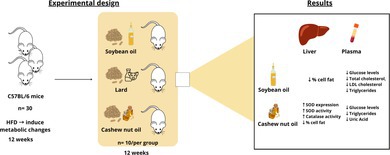

**Abbreviations**
CAT: catalase
FAME: fatty acid methyl esters
GC: gas chromatograph
GLUT: glucose transporter
HDL: high‐density lipoprotein
IRS‐1: insulin receptor substrate 1
LDL: low‐density lipoprotein
MASLD: metabolic dysfunction‐associated steatotic liver disease
MDA: malondialdehyde
mRNA: messenger RNA
MS: mass spectrometer
MUFA: monounsaturated fatty acids
NRF2: nuclear factor erythroid 2‐related factor 2
PBS: phosphate buffer solution
PUFA: polyunsaturated fatty acids
SD: standard deviation
SFA: saturated fatty acids
SOD: superoxide dismutase
TGL: triacylglycerides
VLDL: very low density lipoprotein

## 1 Introduction

Chronic consumption of a high‐fat diet provides higher dietary energy density, elevated serum free fatty acids, and expanded adipose tissue. The excessive accumulation of white adipose tissue is associated with a range of metabolic abnormalities that predispose individuals to the development of conditions such as cardiovascular diseases and insulin resistance (Kojta et al. 2020; Liput et al. 2021; Pavlisova et al. 2016). The role of dietary fatty acids on the body metabolism is not related only to their quantity; their chain lengths, degree of unsaturation, and the configuration of double bonds can also impact their metabolic effects (Pavlisova et al. 2016).

Studies comparing the obesogenic and metabolic effects of different types of dietary lipids suggest that diets rich in saturated fatty acids (SFA) lead to greater increases in hepatic and/or visceral fat compared to diets enriched with polyunsaturated fatty acids (PUFA) (Crescenzo et al. 2015; Wang et al. 2013). Excessive consumption of SFAs can contribute to the release of pro‐inflammatory cytokines, reduce bacterial diversity, and disrupt the intestinal environment. Fatty acids such as palmitic and stearic acids have been shown to exert pathological effects on the intestinal epithelium, gut microbiota, and inflammatory markers (Jamar and Pisani 2023). In contrast, PUFA play essential physiological roles in the body but are susceptible to oxidative damage due to the presence of double bonds (Liput et al. 2021). Additionally, a higher intake of monounsaturated fatty acids (MUFAs) from plant sources has been associated with a reduced risk of type 2 diabetes (Chen et al. 2025) and coronary heart disease risk (Zong et al. 2018). A meta‐analysis showed that greater MUFA consumption may lower the risk of both total and hemorrhagic stroke (Mehrabani et al. 2025). The consensus on the prevention of metabolic dysfunction‐associated steatotic liver disease (MASLD) through dietary modifications (Zeng et al. 2024) recommends increasing the intake of liquid vegetable oils rich in unsaturated fatty acids, fish, whole grains, and vegetables while reducing saturated and trans fats, processed meats, and ultraprocessed foods. Excessive consumption of saturated fatty acids, relative to unsaturated ones, may promote fat accumulation in the liver and exacerbate insulin resistance in individuals with MASLD. Therefore, it is recommended to use liquid vegetable oils for food preparation instead of saturated, hydrogenated, or animal fats. Oils such as olive, canola, corn, and fish oil are rich in mono‐ and polyunsaturated fatty acids, which help reduce plasma LDL cholesterol, increase HDL cholesterol, and contribute to decreased hepatic fat accumulation by reducing endogenous lipid synthesis in the liver.

Cashew nuts ( *Anacardium occidentale* L.) are among the most produced and consumed nuts globally (INC International Nut and Dried Fruit Council 2023). Oil extraction from cashew nuts adds value to the cashew production chain, as they are a good source of edible oil (de Carvalho et al. 2018). Cashew nut oil contains high levels of monounsaturated fatty acids (MUFA) (≈68%) and polyunsaturated fatty acids (PUFA) (≈18%), such as oleic and linoleic acids, respectively, in addition to phenolic compounds (≈2.25 mg gallic acid equivalents/100 g sample), vitamin E, tocopherols, and tocotrienols, which have anti‐inflammatory effects (Meneguelli et al. 2024). This composition may give the oil promising potential as a new value‐added product, attributable to its nutritional benefits and chemical stability (de Carvalho et al. 2018; Leal et al. 2023). Thus, in the present study, we aimed to investigate the effects of adding a vegetable oil obtained from cashew nuts to the diets of C57BL/6J mice subjected to the metabolic effects of a high‐fat diet, comparing two lipid sources: one of vegetable origin (soybean oil) and one of animal origin (lard).

## 2 Methodology

### 2.1 Raw Materials and Preparation of Experimental Diets

The cashew nut oil was produced and donated by the Brazilian Agricultural Research Corporation (Embrapa Agroindústria Tropical, Fortaleza, Brazil). All the following procedures were performed at Embrapa, as previously reported (Leal et al. 2023). Briefly, oil samples were extracted using centrifugation. For sample preparation, the cashew nuts were roasted at 110°C for 15 min in a forced convection air oven (Marconi MA035, Brazil), then ground in a food processor. Water was added in a 4:1 ratio (cashew nuts: water, w/w), and the mixture was homogenized in a Robot coupe food processor at 70°C–80°C for 10 min. This mixture was then centrifuged for 30 min at 4415 × g at room temperature. After centrifugation, the oil was heated in an oven at 105°C for 1 h. The oil samples were stored in 50 mL glass bottles at −80°C to avoid degradation and microorganism proliferation until the experimental tests. The cashew nut oil was characterized as previously described by Meneguelli et al. (2024), including analyses of moisture, ash, protein, lipids, carbohydrates, dietary fibers, amino acids, minerals, vitamin E and its derivatives, total phenolics, antioxidant capacity, fatty acid profile, acidity level, and peroxide index. The raw materials were sourced from the same harvest. The soybean oil and lard were commercially obtained.

The standard diet provided to the animals was commercially obtained (Labina, Paulínia, São Paulo, Brazil). For the preparation of the experimental diets, the commercially obtained diet was ground in an industrial mill to form a powder, allowing the incorporation of oils and lard and the subsequent production of pellets. According to the experimental group design, soybean oil was added to the diet of the control group, lard was added to the diet of test group 1, and cashew nut oil was added to the diet of test group 2, all at a proportion of 5% per kg of diet. The diets were prepared weekly, packed, stored in the dark, and frozen at −20°C until offered to the animals.

### 2.2 Fatty Acid Profile Analysis of the Diets

Lipid extraction from the diet was performed according to the Bligh and Dyer method (Bligh and Dyer 1959). Briefly, 10 g of the diet was mixed with methanol, chloroform, and water (2:1:0.8) and stirred for 20 min. Then, chloroform and a 1.5% sodium sulfate (Na_2_SO_4_) solution were added and stirred for an additional 2 min. Finally, the samples were filtered using filter paper containing Na_2_SO_4_, and the solvent was cold‐evaporated using nitrogen.

The fatty acid profile of the diet was determined after preparation of fatty acid methyl esters (FAME) according to the International Olive Council (Zeng et al. 2013). Briefly, the diet was dissolved in hexane, and methanolic potassium hydroxide (0.2 mL, 2 M) was added and mixed for 30 min. After this period, the sample was left to stand for 10 min, and the organic phase was collected in a 2 mL vial for analysis by gas chromatography. The results were expressed based on the normalization of the areas.

An Agilent 7820A gas chromatograph (GC) (Palo Alto, CA, USA) equipped with a G4513A autosampler and coupled to an Agilent 5975 quadrupole grid mass spectrometer (MS) was used. The carrier gas used was helium (99.9992% purity). Mass spectra were obtained by scanning (50–550 Da). The chromatographic results were recorded in the Agilent MassHunter B.07.00 qualitative analysis software. The composition of the FAMEs was characterized by comparison with the NIST/EPA/NIH Mass Spectral Library databases (version 2.2, 2014) (Palacio de Araujo et al. 2023).

### 2.3 Experimental Design

Thirty C57BL‐6 ( *Mus musculus* ) male mice at four weeks old, weighing approximately 22 g, were obtained from the Central Animal Facility of the Health and Biological Sciences Center at the Federal University of Viçosa (UFV). The experimental protocol was approved by the Animal Experimentation Ethics Committee (CEUA) of the Federal University of Viçosa (MG), under process number 37/2022, in accordance with current legislation (Law No. 11,794, of October 8, 2008).

The sample size was calculated according to Mera et al. (1998) (Mera et al. 1998), based on body weight loss and no animals were excluded. A confidence level of 95% was used (*α* < 0.05). After 4 weeks of acclimatization, prior to the dietary intervention, mice were weighed, stratified according to their body weight, and assigned to the cages to ensure comparable mean baseline body weights across the cages. The animals were housed collectively in polyethylene cages (5 animals/cage) during the entire experiment. The animals were kept in a room with controlled temperature (22°C ± 2°C), humidity (60%–70%), and a 12 h light/dark cycle. In the first 12 weeks, all animals received a high‐fat diet (45% of fat) to induce the metabolic effects. For the preparation of the high‐fat diet, 213 g of lard per kg of diet was mixed with the commercial powdered diet (which contains 40 g of lipids per kg of diet), resulting in a formulation providing approximately 45% of total calories from fat, and pellets were then reconstituted. After this period, the animals were relocated into three experimental groups (*n* = 10/group). They were weighed, stratified according to their body weight, and then randomized into diet groups to achieve similar average body weights among groups. The animals received the commercial diet (40 g of lip/kg of diet) added with one of the lipid sources tested for 12 weeks: Soybean oil (standard commercial diet added with 5% of soybean oil), Lard (standard commercial diet added with 5% of lard), or Cashew nut oil (standard commercial diet added with 5% of cashew nut oil), which therefore represents a low‐fat diet compared to the induction diet. Water and diet were provided ad libitum during the experimental period. No animals were lost throughout the experiment. The researchers were not blind.

Body weight and food consumption were monitored weekly on a fixed day during the experimental period. The animals were weighed individually. Food intake was measured on a per‐cage basis. During the 12‐week experiment, weekly food intake was recorded separately for each cage. At the end, the weekly consumption values from all cages and weeks were averaged to obtain the final weekly food intake per group. Blood glucose levels were measured at baseline and at the end of the experimental period in the tail vein. Briefly, a small cut was made at the end of the animal's tail and blood glucose levels were measured with a portable glucometer (Accu‐Chek, Roche) using appropriate test strips.

At the end of the experiment, after 8 h fasting, the animals were individually placed in a transparent sealed box for anesthetic saturation, 3–5 min of isoflurane (Isoflorine, Cristalia, Itapira, Brazil), using a simple circuit with a flowmeter coupled to an oxygen cylinder (3%–5% mixture of isoflurane and oxygen). Physiological parameters and reflexes were evaluated to determine the degree of anesthesia sensitivity, minimizing the stress of manipulation. The animals were euthanized by total exsanguination from the abdominal artery; blood was collected, centrifuged, and the plasma was stored at −80°C. The liver was removed, washed in phosphate buffer solution (PBS), weighed, and part was stored at −80°C for later analysis, and part was fixed in formalin for 24 h and kept in 70% ethanol for histological analysis.

### 2.4 General Parameters and Consumption

The Lee index was calculated as the cube root of body weight (in grams) divided by the nose‐to‐anus length (in centimeters) (Novelli et al. 2006). The abdominal perimeter was obtained at the midpoint between the skull and the pelvic limbs of the animal (Reynés et al. 2014). The hepatic index was defined as the percentage of liver weight relative to the body weight (Kim et al. 2016). Food intake was determined based on the amount (g) of diet offered by subtracting leftovers (g) not ingested. The quantification of leftovers was performed weekly on an electronic scale (Marte Slim, model M 2K, São Paulo, Brazil).

### 2.5 Metabolic Markers Assessment

Plasma concentrations of glycemia, total cholesterol, high‐density lipoprotein (HDL) cholesterol, low‐density lipoprotein (LDL) cholesterol, triacylglycerides (TGL), creatinine, and uric acid concentrations were measured by colorimetric methods using commercially available kits (Bioclin, Belo Horizonte, Brazil). Analyses were conducted on a BS‐200 chemistry analyzer (Bioclin) at the Laboratory of Clinical Analysis and Genomics of the UFV.

### 2.6 Oxidative Stress in the Liver Tissue

#### 2.6.1 Homogenate Preparation

To obtain a liver homogenate, 100 mg of liver sample was mixed with 1000 μL of phosphate buffer (50 mM). The sample was macerated and centrifuged at 12000 rpm and 4°C for 10 min. The supernatant was removed and stored in an ultrafreezer until the time of analysis. Total protein in the liver homogenate was quantified by the Lowry method (Lowry et al. 1951).

#### 2.6.2 Superoxide Dismutase (SOD) Quantification and Catalase (CAT) Activity

Quantification of SOD was determined based on the ability of this enzyme to reduce the auto‐oxidation of pyrogallol. The reading was performed at 570 nm. CAT activity was determined by its ability to cleave hydrogen peroxide (H_2_O_2_) into water and molecular oxygen. Absorbance was determined at 0, 30, and 60 s, at 240 nm. All readings were performed in a spectrophotometer (Multiskan GO, Thermo Scientific, Ratastie, Finland), and the results expressed in units per milligram of protein.

#### 2.6.3 Malondialdehyde (MDA)

Lipid peroxidation was evaluated based on the reaction capacity between thiobarbituric acid and MDA (peroxidation product). The reading was performed at 540 nm. The MDA concentration was calculated using the molar absorptivity coefficient *ε* = 1.56 × 10^5^ M^−1^ cm^−1^. The results were expressed in moles of MDA per milligram of protein.

### 2.7 Extraction of mRNA From Liver Tissue and cDNA Synthesis

Total RNA was isolated using TRIzol reagent (Ambion, Life Technologies, USA) under RNase‐free conditions. Briefly, approximately 50 mg of frozen liver tissue was homogenized in 1 mL of TRIzol, followed by phase separation with chloroform, RNA precipitation with isopropanol, and washing with 75% ethanol. To synthesize the cDNA, the GoScript Reverse Transcription System (Promega, A5001, USA) was used, following the manufacturer's instructions.

### 2.8 Determination of Gene Expression of NRF2 and SOD by Quantitative Reverse Transcriptase Polymerase Chain Reaction (RT‐qPCR)

The liver mRNA gene expression was analyzed by RT‐qPCR. The SYBR Green PCR master mix from Applied Biosystems (Foster City, CA) was employed, and the analyses were performed at StepOne Real‐Time PCR System (Thermo Fisher Scientific) using the SYBR‐Green fluorescence quantification system and Primer Express software system, v3.0.1 (Applied Biosystems, Foster City, CA, USA). Follow and reverse primer sequences (Choma Biotechnologies) were used to Nuclear Factor Erythroid 2‐Related Factor 2 (NRF2) and Superoxide Dismutase (SOD) (Table 1). The relative expression levels of mRNA were normalized using β‐actin. All the steps were performed under free RNase condition.

**TABLE 1:** Sequence of primers used in the RT‐PCR analysis.

Gene	Primer sequences	
NRF2	Forward (5′–3′)	GCCCTCAGCATGATGGACTT
	Reverse (5′–3′)	AACTTGTACCGCCTCGTCTG
Zn‐SOD1	Forward (5′–3′)	GAGCAGAAGGCAAGCGGTGAA
	Reverse (5′–3′)	CCACATTGCCCAGGTCTG
β‐actin	Forward (5′–3′)	TTCGTTGCCGGTCCACACCC
	Reverse (5′–3′)	GCTTTGCACATGCCGGAGCC

### 2.9 Histological Analysis in the Liver Tissue

The liver tissue samples were fixed in 10% formaldehyde and embedded in paraffin. The blocks were sliced with semi‐serial sections (5 μm thickness) and stained by the hematoxylin/eosin technique. The analyses were performed and photographed under a bright‐field microscope (Primo Star; Carl Zeiss Microscopy, GmbH, Germany) in a 20× objective. Ten liver fields were selected for each animal, with each histological field containing a 266‐point reticulum on the images until (Cupertino et al. 2013). The images of the histological sections were analyzed using the Image J V 1.8.0 software system (National Institute of Health, USA).

### 2.10 Statistical Analysis

The experiments were conducted in a completely randomized design with 10 biological replicates (*n* = 10 animals per group). All the results were expressed as means ± standard deviation (SD). Differences were considered significant when *p* < 0.05. Outliers have been excluded. The Shapiro–Wilk normality test was used to evaluate values for normal distribution and variance homogeneity. Normally distributed results were analyzed using a one‐way analysis of variance (ANOVA), followed by the post hoc Tukey test. The variables without normal distribution were analyzed using Kruskal–Wallis and a post hoc Dunn's test. Paired *t*‐tests were used to compare differences between baseline data and final data. The correlation between the general parameters, metabolic markers and oxidative stress biomarkers, and histological parameters was analyzed by Pearson's correlation test. The variables that did not show a normal distribution were transformed using the logarithmic scale. The statistical analyses and graphs were performed using the statistical software GraphPad Prism software, version 10.1.2 (GraphPad Software Inc., San Diego, CA, USA).

## 3 Results

### 3.1 Fatty Acid Profile of the Experimental Diets

The diet added with cashew nut oil presented the oleic acid (C18:1 n‐9) as its main fatty acid (50.21%), followed by linoleic acid (C18:2 n‐6, n‐9) (24.40%) and palmitic acid (C16:0) (11.74%). The diet added with soybean oil presented the linoleic acid as its main fatty acid (44.14%), followed by oleic acid (27.72%) and palmitic acid (13.01%). The diet added with lard presented palmitic acid as its main fatty acid (35.06%), followed by linoleic acid (33.32%) and stearic acid (C18:0) (20.98%). Oleic acid, a monounsaturated fatty acid, was present in both the cashew nut oil and soybean oil groups, with higher levels in the cashew nut oil group. Linoleic acid, a polyunsaturated fatty acid, was highest in the soybean oil group, followed by the lard group and then the cashew nut oil group. Palmitic and stearic acids, saturated fatty acids, were highest in the lard group. The lipid composition was more diverse in the group fed lard, followed by the group fed soybean oil, and lastly, the group fed cashew nut oil (Table 2).

**TABLE 2:** Fatty acid profile of experimental diets.

Fatty acid	Diet					
	Soybean oil		Lard		Cashew nut oil	
	Mean	SD	Mean	SD	Mean	SD
C14:0	0.10^b^	0.019	1.97^a^	0.119	0.05^b^	0.003
C15:0	—		0.09	0.005	—	
C16:0	13.01^b^	0.612	35.06^a^	0.757	11.74^b^	0.240
C17:0	0.13^b^	0.019	0.55^a^	0.018	0.16^b^	0.006
C18:0	5.13^c^	0.705	20.98^a^	0.311	9.54^b^	0.090
C20:0	0.52^b^	0.095	0.45^b^	0.019	0.71^a^	0.014
C22:0	0.55^a^	0.087	0.12^c^	0.008	0.22^b^	0.002
C23:0	0.15	0.145				
C24:0	0.24^a^	0.008	0.08^b^	0.028	0.21^a^	0.003
C16:1 n‐7	0.13^c^	0.022	2.56^a^	0.032	0.33^b^	0.010
C17:1 n‐7	0.07	0.017	0.33*	0.024	—	
C17:1 n‐8	—		—		0.07	0.001
C18:1 n‐5	—		—		0.66	0.005
C18:1 n‐7	1.56	0.035	—		—	
C18:1 n‐9	27.72	0.447	—		50.21*	0.401
C20:1 n‐9	0.33^b^	0.054	0.99^a^	0.058	0.32^b^	0.023
trans‐C18:2 n‐6	—		0.09	0.020	—	
cis‐C18:2 n‐6, n9	44.14^a^	2.153	33.32^b^	0.434	24.40^c^	0.241
C18:3 n‐3	6.04^a^	0.441	2.23^b^	0.172	1.36^c^	0.016
C20:2 n‐6	0.11	0.084	0.79*	0.060	—	
C20:3 n‐6	—		0.09	0.008	—	
C20:4 n‐3	0.07	0.002				
C20:4 n‐6	—		0.28	0.023	—	
Total saturated fatty acids	19.84^c^	1.39	59.31^a^	0.47	22.64^b^	0.19
Total monounsaturated fatty acids	29.81^b^	0.51	3.88^c^	0.04	51.59^a^	0.40
Total polyunsaturated fatty acids	50.36^a^	1.81	36.80^b^	0.46	25.76^c^	0.22

*Note:* Values are percentage and represented as means ± standard deviation (SD) of triplicates. Fatty acids found in all three diets were compared using one‐way ANOVA followed by a post hoc Tukey test, while those found in only two types of diets were compared using the *t*‐test. ^a, b, c^ In the same row not indicated by the same letter are significantly different (*p* < 0.05) according to one‐way ANOVA followed by post hoc Tukey test.

* Means statistically significant difference according to the *t*‐test (*p* < 0.05).

### 3.2 General Parameters and Food Consumption

The final weight and Lee index were not different among the experimental groups. Also, the measurements of abdominal perimeter (initial, final, and delta) and hepatic index were not different among the groups. The weekly food consumption was higher in the soybean oil group compared with both other groups (Table 3, Section A).

**TABLE 3:** Effects of soybean oil, lard, or cashew nut oil for 12 weeks on general parameters and metabolic variables in C57BL‐6 mice.

	Soybean oil		Lard		Cashew nut oil	
	Mean	SD	Mean	SD	Mean	SD
*Section A: General parameters and food consumption*						
Final weight (g)	30.13^a^	2.55	29.49^a^	3.89	30.25^a^	3.42
Lee index	0.38^a^	0.01	0.38^a^	0.02	0.38^a^	0.01
Initial abdominal perimeter (cm)	7.36^a^	0.21	7.42^a^	0.30	7.54^a^	0.60
Final abdominal perimeter (cm)	6.80^a^	0.36	6.66^a^	0.40	6.5^a^	0.38
Δ abdominal perimeter (cm)	−0.56^a^	0.48	−0.76^a^	0.53	−1.00^a^	0.86
Hepatic index	5.26^a^	0.62	5.27^a^	0.50	5.44^a^	0.42
Group weekly food consumption (g)	243.80^a^	21.32	219.30^b^	22.68	217.38^b^	22.47
*Section B: Metabolic variables*						
Final glycemia (mg/dL)	139.30^b^	13.82	165.20^a^	28.07	135.30^b^	15.03
Total cholesterol (mg/dL)	44.29^b^	3.82	51.57^a^	6.70	46.57^ab^	4.08
LDL cholesterol (mg/dL)	17.29^b^	2.43	24.83^a^	5.15	20.71^ab^	2.81
HDL cholesterol (mg/dL)	27.71^a^	4.79	28.86^a^	4.71	24.71^a^	1.80
Triglycerides (mg/dL)	47.14^b^	14.22	66.57^a^	16.68	43.43^b^	4.96
Creatinine (mg/dL)	0.33^a^	0.03	0.35^a^	0.04	0.34^a^	0.03
Uric Acid (mg/dL)	2.41^ab^	0.31	3.54^a^	1.52	1.86^b^	0.29

*Note:* Values are means ± standard deviation (SD). *n* = 10/group general parameters and *n* = 7/group for metabolic variables. Δ abdominal perimeter (Final abdominal perimeter ‐ Initial abdominal perimeter). Hepatic index: liver weight/body weight × 100. The data about weekly food consumption are represented by means of food consumption pool of each group (1 pool/week). LDL, Low‐density lipoprotein; HDL, high‐density lipoprotein. ^a‐b^ Different letters in the same row indicated statistical difference (*p* < 0.05) according to one way ANOVA followed by post hoc Tukey test (parametric data) or Kruskal–Wallis followed by Dunn's test (non‐parametric data).

### 3.3 Metabolic Markers

**FIGURE 1:**
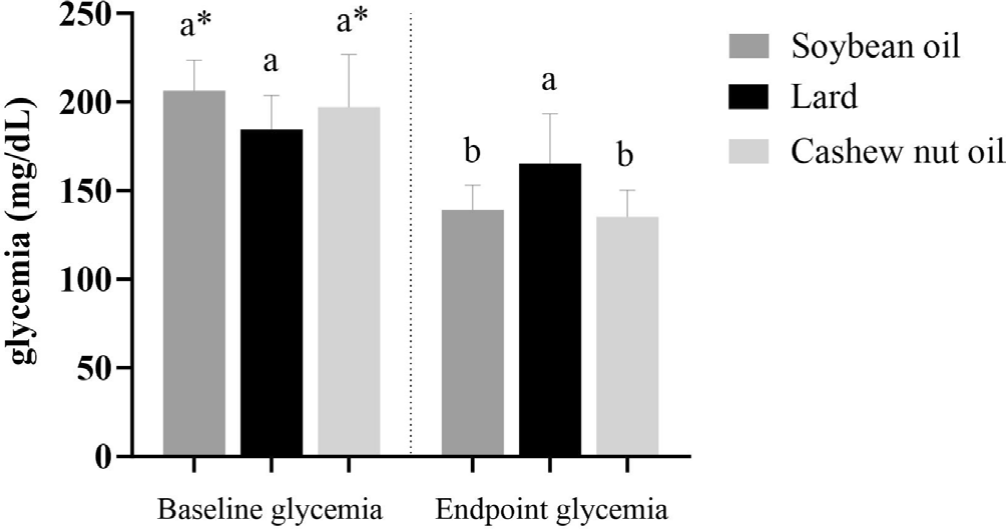
Glycemia at baseline and endpoint. Values are means ± SD. *n* = 10/group. Baseline glycemia corresponds to the beginning of the intervention, after the consumption of the high‐fat diet. Endpoint glycemia corresponds to the end of the intervention. ^a‐b^ different letters in the same row indicate statistical difference (*p* < 0.05) according to one‐way ANOVA followed by post hoc Tukey test. * Means statistically significant difference between baseline and endpoint values in the same group, according to the paired *t*‐test (*p* < 0.05).

Glycemia before the treatment was not different among the experimental groups. Endpoint glucose levels were higher in the lard group compared to the soybean oil and cashew nut oil groups, but with no difference regarding baseline value. The groups that received soybean oil and cashew nut oil demonstrated similar endpoint glucose values, but they were lower compared with their baseline values (Figure 1).

The mice fed soybean oil had lower total cholesterol and LDL cholesterol compared with the lard‐fed group, while the cashew nut oil group had similar total cholesterol and LDL cholesterol levels to the lard‐ and soybean oil‐fed groups. The soybean oil‐ and cashew nut oil‐fed groups had lower triglyceride and glycemia levels compared with the lard‐fed group. Cashew nut oil also reduced uric acid levels compared with the lard‐fed group. HDL cholesterol and creatinine levels did not differ among the three diet groups (Table 3, Section B).

### 3.4 Oxidative Stress in the Liver

**FIGURE 2:**
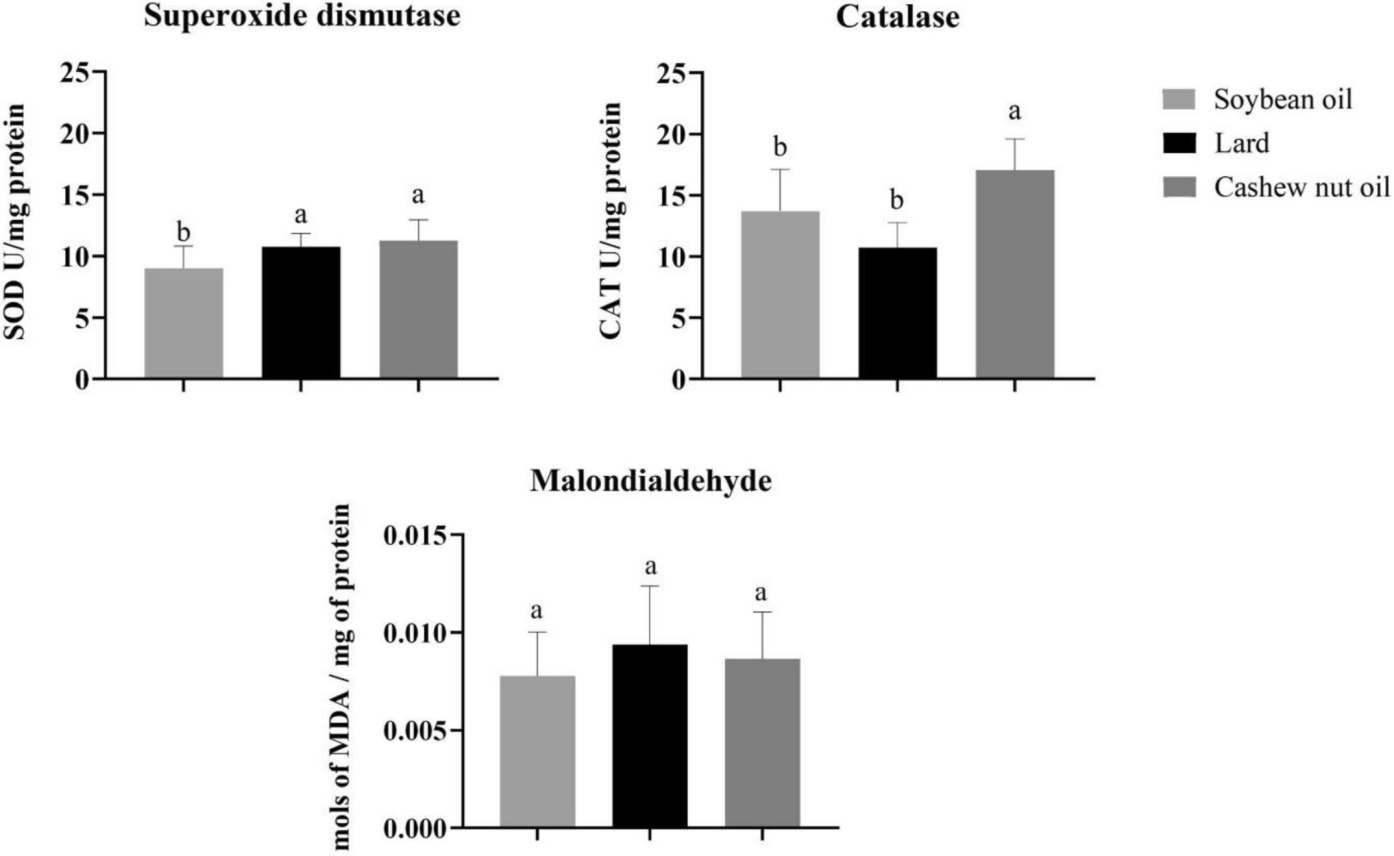
Oxidative stress analysis. (a) Activity of SOD expressed as Units of SOD/mg of protein; (b) Activity of CAT expressed as Units of catalase/mg of protein; (c) mols of malondialdehyde/mg of protein. CAT, catalase; MDA, malondialdehyde; SOD, superoxide dismutase. Values are means ± SD. *n* = 10/group. CAT, catalase; SOD, superoxide dismutase; U, units. ^a‐b^ treatment group means not indicated by the same letter are significantly different (*p* < 0.05) according to one‐way ANOVA followed by post hoc Tukey test (*p* < 0.05).

The SOD activity was similar between animals fed lard and cashew nut oil, and it was higher than the group fed soybean oil. The activity of catalase increased in the group fed cashew nut oil compared to both other groups (lard and soybean oil). The levels of malondialdehyde did not differ among the groups (Figure 2).

### 3.5 Gene Expression Analysis

**FIGURE 3:**
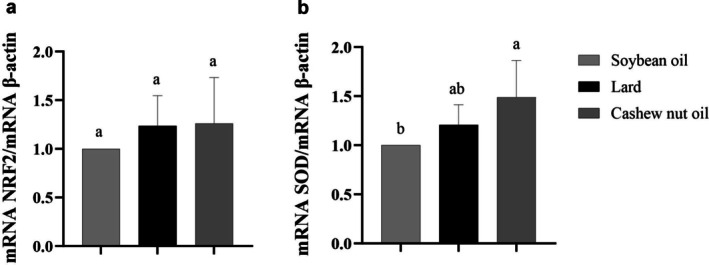
Effect of soybean oil, lard, or cashew nut oil for 12 weeks on NRF2 and SOD gene expression in liver. RT‐qPCR analysis. Values represent means ± standard deviation. (a) NRF2 expression; (b) SOD expression. NRF2, nuclear factor erythroid 2‐related factor 2; SOD, superoxide dismutase. Means followed by the same lowercase letter did not differ significantly according to the Tukey test at 5% probability.

The expression levels of NRF2 did not differ among the experimental groups. However, cashew nut oil consumption did promote a higher SOD mRNA expression compared to soybean oil, and lard consumption did not differ in SOD expression compared to soybean oil and cashew nut oil (Figure 3).

### 3.6 Liver Histological Analysis

**FIGURE 4:**
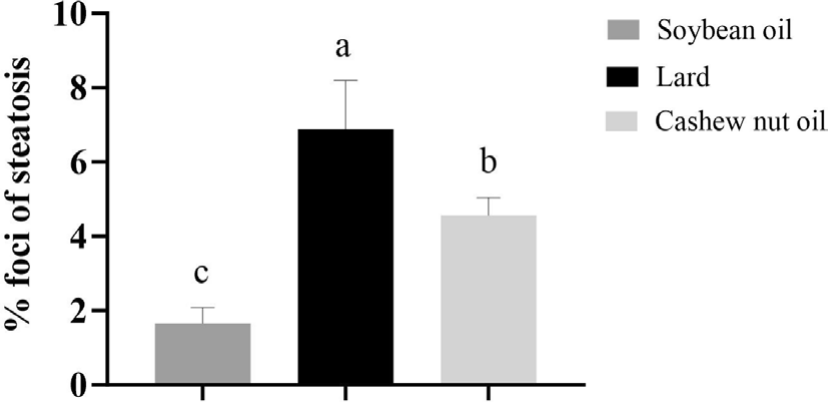
Effect of soybean oil, lard, or cashew nut oil for 12 weeks on percentage of foci of microvesicular steatosis in the liver. Values are means ± SD. *n* = 7/group. ^a‐b^ treatment group means not indicated by the same letter are significantly different (*p* < 0.05) according to one‐way ANOVA followed by post hoc Tukey test (parametric data).

**FIGURE 5:**
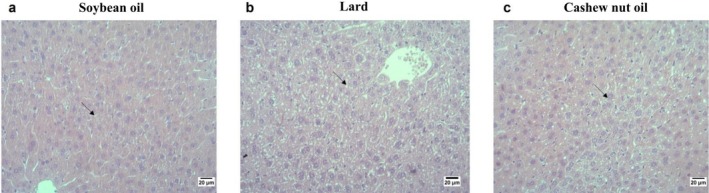
Histological images of the effect of soybean oil, lard or cashew nut oil for 12 weeks on percentual of foci of microvesicular steatosis in the liver. (a) Group fed with soybean oil; (b) Group fed with lard; (c) Group fed cashew nut oil. Staining was carried out with hematoxylin and eosin. Objective: 20×. Black arrows indicate foci of microvesicular steatosis. Scale bar = 20 μm.

The animals in the soybean oil group demonstrated a lower number of foci of microvesicular steatosis compared to the groups fed with lard or cashew nut oil (Figures 4 and 5). Moreover, the animals fed with cashew nut oil demonstrated a lower number of foci of microvesicular steatosis compared to the group fed with lard.

### 3.7 Pearson Correlation Analysis

**FIGURE 6:**
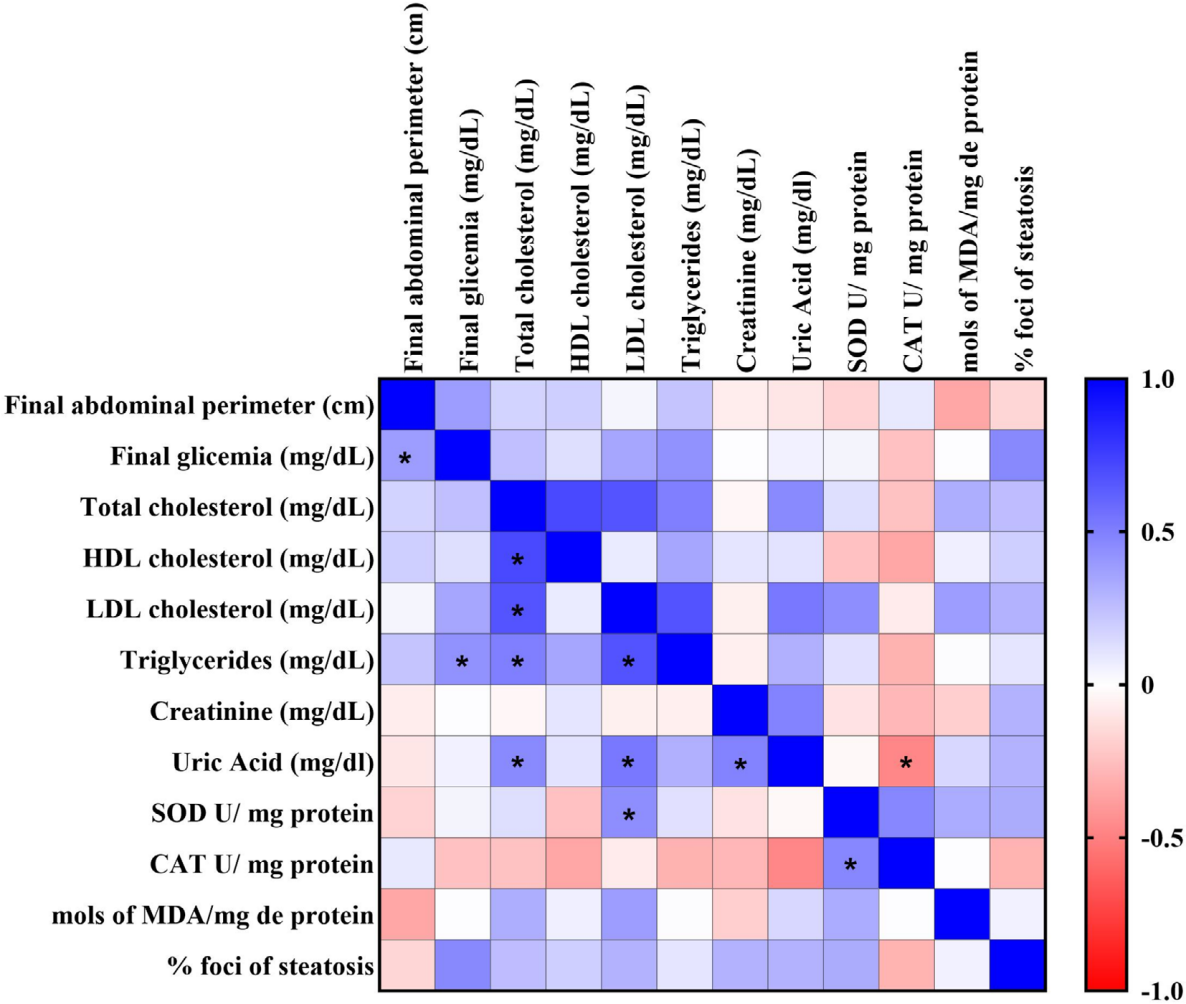
Heatmap of Pearson correlation analysis. CAT, catalase; HDL, high‐density lipoprotein; LDL, low‐density lipoprotein; MDA, malondialdehyde; SOD, superoxide dismutase. * Means statistically significant difference (*p* < 0.05).

Pearson correlation analysis was used to evaluate the relationship between changes in general parameters, metabolic markers, oxidative stress biomarkers, and histological parameters. Positive correlations were observed between blood glucose and triglycerides, total cholesterol and LDL, total cholesterol and HDL, total cholesterol and triglycerides, total cholesterol and uric acid, LDL and triglycerides, LDL and uric acid, LDL and SOD, SOD and CAT (Figure 6).

## 4 Discussion

The quantity and quality of fat consumed in the diet can contribute to excess weight and associated comorbidities (Piccinin et al. 2019). Cashew nut oil is a source of monounsaturated and polyunsaturated fatty acids and phenolic compounds (Meneguelli et al. 2024), providing added value to this Brazilian oilseed. However, the effects of this oil on the metabolism of animals subjected to the effects of consuming a high‐fat diet have not yet been investigated. Currently, cashew nut oil is not commercially available in Brazil, which makes it an interesting prospect for the industry, especially considering its favorable nutritional composition. In the present study, the fatty acids found in the highest quantities in the diet added with cashew nut oil were oleic (monounsaturated), followed by linoleic acid (polyunsaturated), which have also been reported by our research group as the predominant fatty acids in cashew nut oil (Leal et al. 2023; Meneguelli et al. 2024). Furthermore, the group fed with cashew nut oil showed lower levels of glycemia, triglycerides, uric acid, and hepatic foci of microvesicular steatosis, in contrast to what was observed in the group fed with a diet added with lard, which had higher levels of palmitic acid and presented higher glycemia, serum lipids, uric acid, and hepatic foci of microvesicular steatosis.

Oleic acid has anti‐inflammatory and hypocholesterolemic properties (Piccinin et al. 2019; Santa‐María et al. 2023), and linoleic acid is a component of phospholipids in cell membranes (Christi and Harwoo 2020). Furthermore, oleic acid, present in cashew nut oil, can prevent the increase in the synthesis of complex lipids induced by palmitic acid, in addition to attenuating its deleterious effects by increasing the mitochondrial oxidation of saturated fatty acids and promoting their accumulation in the form of triacylglycerol, thus reducing the synthesis of diacylglycerol and ceramide (Palomer et al. 2018).

The consumption of a diet added with soybean oil, with a predominance of linoleic acid, presented lower plasma levels of total cholesterol, LDL‐cholesterol, and triglycerides. According to the study by Yang et al. (2022), animals fed a diet added with conventional soybean oil also showed a reduction in plasma lipids (total cholesterol, very low‐density lipoprotein [VLDL], and LDL). The authors attributed the hypolipidemic effects partially to the downregulation of hepatic *Srebp2* mRNA expression and the upregulation of *Cyp7a1*, which encodes the key enzyme in the conversion of cholesterol into bile acids, in addition to the improvement in hepatic clearance of circulating LDL.

Here, the group fed with cashew nut oil showed lower levels of plasma uric acid. This is a metabolic marker, produced by the catabolism of purine nucleotides, and can be modulated by dietary patterns (Liu et al. 2015). It has been associated with steatotic liver disease and metabolic dysfunction, since high concentrations of uric acid induce the accumulation of reactive oxygen species in hepatocytes, leading to mitochondrial damage (Hu et al. 2018; Yang et al. 2016) with a pro‐oxidant and pro‐inflammatory role (Sirota et al. 2013). Thus, elevated uric acid may be one of the metabolic changes associated with the consumption of a high‐fat diet. Metabolic markers such as reduced blood glucose, total cholesterol, triglycerides, and uric acid, together, reduce the risk of comorbidities associated with the consumption of a high‐fat diet (Borghi et al. 2022). A study evaluating an extract rich in polyunsaturated (arachidonic acid) and monounsaturated (oleic acid) fatty acids found that its administration reduced serum uric acid in hyperuricemic rats. The anti‐hyperuricemic activity could be associated with the ability of fatty acids to regulate the expression of urate transporters, which are renal urate reabsorbers—GLUT9a and GLUT9b (Ikhsan et al. 2023). Furthermore, Li et al. (2019) observed that celery seed oil extract has suppressive effects on uric acid in mice with hyperuricemia. These effects were attributed, in part, to the inhibition of oxidative stress. The extract is rich in flavonoids, which have been shown to inhibit xanthine oxidase activity, increase SOD and glutathione peroxidase production, and reduce reactive oxygen species levels. Xanthine oxidase is a key enzyme in the conversion of xanthine and hypoxanthine to uric acid, a process that also generates reactive oxygen species. In our study, the consumption of a diet added with cashew nut oil, after a high‐fat diet, reduced uric acid, an effect associated with both a decrease in hepatic fat and a reduction in oxidative stress, evidenced by increased activity of the antioxidant enzyme catalase, as observed in our correlation analysis.

In the antioxidative process, SOD is the first line of defense among antioxidant enzymes against oxygen free radicals, catalyzing the dismutation of superoxide anion into oxygen (O_2_) and hydrogen peroxide (H_2_O_2_). After activation of SOD and production of hydrogen peroxide by this enzyme, activation of the second line of antioxidant defense, the production of catalase, is necessary. Catalase is an enzyme whose function is the dismutation of H_2_O_2_ into oxygen and water, acting in cellular defense against oxidative damage by H_2_O_2_. The groups fed lard and cashew nut oil exhibited similar SOD activity, indicating a comparable first‐line defense against superoxide radicals. Both groups needed to increase the production of antioxidant enzymes to combat reactive oxygen species and activate the second line of antioxidant defense, catalase. However, only the cashew nut oil group showed an increase in catalase activity, suggesting a more effective activation of the second line of antioxidant defense to neutralize hydrogen peroxide. Lard, despite inducing similar SOD activity, did not elicit a corresponding increase in catalase, indicating a limited enhancement of the downstream antioxidant response. At the gene expression level, SOD mRNA was higher in the cashew nut oil group compared to soybean oil, while lard did not differ from either group, supporting the enhanced enzymatic response observed in cashew nut oil‐fed animals. NRF2 expression did not differ among the groups, indicating that the modulation of SOD and catalase activities by cashew nut oil may occur independently of changes in NRF2 transcription. The beneficial effect observed with cashew nut oil may not be solely attributed to its fatty acid profile, but also to its content of total phenolics and other antioxidant compounds, as previously characterized by our research group (Meneguelli et al. 2024), which likely contribute to its capacity to modulate antioxidant defenses.

In our study, plasma concentrations of glucose, total cholesterol, LDL‐cholesterol, and triglycerides at the end of the study were higher in the group that received a diet with added lard, which showed a predominance of palmitic acid, a saturated fatty acid. Palmitic acid attenuates the insulin signaling pathway through mechanisms that lead to insulin resistance. One of the mechanisms is mediated by the increase in plasma non‐esterified fatty acids, favoring the rate of delivery of fatty acids to non‐adipose tissues, such as the liver, muscle, heart, and pancreas. This ectopic deposition of lipids promotes dysfunction and cell death. Thus, increased intracellular levels of palmitic acid exceed its mitochondrial oxidation and can be converted into deleterious complex lipids derived from fatty acids, such as diacylglycerol and ceramide, which activate mediators that attenuate the insulin signaling pathway by phosphorylating serine residues in insulin receptor substrate 1 (IRS‐1). Thus, the accumulation of free fatty acids in non‐adipose tissues, such as the liver, is implicated in the development of insulin resistance (Lv et al. 2024). In situations of excess energy, triglycerides are stored in adipose tissue, which is also the largest reservoir of cholesterol in the body. The amount of cholesterol and triacylglycerols increases with adipocyte size (Sheikh et al. 2024; Yu et al. 2010). This is consistent with our results, which showed that the group fed the diet with added lard, rich in palmitic acid, had more foci of microvesicular steatosis and higher values of total cholesterol and triglycerides. Furthermore, this is in agreement with the positive correlation found between cholesterol and triglycerides.

In the study by Ortiz et al. (2020), mice fed a HFD developed hepatic mitochondrial dysfunction associated with steatosis. However, this effect was prevented when the HFD was supplemented with docosahexaenoic acid (DHA), an n‐3 polyunsaturated fatty acid (PUFA), combined with hydroxytyrosol. The beneficial effects were attributed, at least in part, to the inhibition of oxidative stress by the antioxidant properties of hydroxytyrosol, as well as to the role of DHA in maintaining mitochondrial function and lipid homeostasis. HFD‐induced mitochondrial dysfunction and oxidative stress are closely linked to an imbalance in transcription factors, leading to a pro‐lipogenic state characterized by upregulation of SREBP and downregulation of PPAR‐α, which together promote hepatic lipid synthesis and reduce fatty acid oxidation (Ortiz et al. 2020). This mechanism is further aggravated by the depletion of n‐3 PUFAs in the liver, such as DHA, which play a crucial role in regulating these pathways. Besides that, diets enriched with rapeseed oil have demonstrated anti‐obesity and hepatoprotective effects by decreasing hepatic fatty acid synthesis and enhancing fatty acid metabolism, effects attributed to its high content of polyphenolic compounds (Zhou et al. 2023). Similarly, other phenolic compounds, such as flavones from *Abelmoschus manihot* (L.) Medik, reduced hepatic lipid accumulation, serum total cholesterol, and triglyceride levels, while attenuating inflammation and oxidative damage in mice with metabolic associated fatty liver disease (Lv et al. 2024). Moreover, resveratrol supplementation in HFD‐fed rats prevented hepatic steatosis, suggesting that its antioxidant and anti‐inflammatory properties are central to its protective effects against hepatic disorders and hyperlipidemia (Yasmin et al. 2025). Taken together, these findings highlight the role of dietary polyphenols in modulating oxidative stress and lipid metabolism, thereby protecting against liver injury induced by HFD. In this way, in our study, the consumption of a diet added with cashew nut oil, after prolonged consumption of a high‐fat diet, was shown to be beneficial in reducing hepatic fat deposition, reducing triglycerides, glycemia, uric acid, and improving antioxidant capacity. These effects are probably attributed to its fatty acid composition, rich in oleic acid, a monounsaturated fatty acid, which acts to reduce hepatic fat deposition, impacting a lower quantity of triglycerides, better insulin signaling, and lower uric acid concentration, and also in its composition, the presence of bioactive compounds such as phenolics that can contribute to the antioxidant capacity of the oil.

## 5 Conclusion

Cashew nut oil, a new plant‐based product, has been shown to reduce blood glucose, triglycerides, uric acid, hepatic foci of microvesicular steatosis, and increase SOD expression and activity and catalase activity. Soybean oil, traditionally used in food, exhibited lowering effects on blood glucose, total cholesterol, LDL cholesterol, triglycerides, and also hepatic foci of microvesicular steatosis. Both oils are rich in unsaturated fatty acids and were compared with the consumption of lard, a source of animal fat and rich in saturated fatty acids. Thus, our results support that the quality of the fatty acid is an important determinant of its metabolic effect. Moreover, we show for the first time the additional benefits of consumption of cashew nut oil, compared to lard fat, which are mediated by the reduction of hepatic fat accumulation and reduction of oxidative stress.

## Author Contributions

Conceptualization: Marcella Duarte Villas Mishima, Andressa Ladeira Bernardes, Carlos Alexandre Pinheiro, Helen Hermana Hermsdorff, and Maria do Carmo Gouveia Peluzio. Methodology: Marcella Duarte Villas Mishima, Andressa Ladeira Bernardes, Carlos Alexandre Pinheiro, Luisa Gouveia Lana, Iasmim Xisto Campos, Valéria Silva de Lana, Izabela Maria Montezano de Carvalho, Patrícia Fontes Pinheiro, and Renata Celi Lopes Toledo. Formal analysis: Marcella Duarte Villas Mishima and Andressa Ladeira Bernardes. Investigation: Marcella Duarte Villas Mishima, Andressa Ladeira Bernardes, Carlos Alexandre Pinheiro, Luisa Gouveia Lana, Iasmim Xisto Campos, Valéria Silva de Lana, Izabela Maria Montezano de Carvalho, Patrícia Fontes Pinheiro, and Renata Celi Lopes Toledo. Resources: Ana Paula Dionísio, Helen Hermana Hermsdorff, and Maria do Carmo Gouveia Peluzio. Data curation: Marcella Duarte Villas Mishima and Andressa Ladeira Bernardes. Writing – original draft preparation: Marcella Duarte Villas Mishima and Andressa Ladeira Bernardes. Writing – review and editing: Ana Paula Dionísio, Helen Hermana Hermsdorff, and Maria do Carmo Gouveia Peluzio. Supervision: Maria do Carmo Gouveia Peluzio. Funding acquisition: Helen Hermana Hermsdorff and Maria do Carmo Gouveia Peluzio.

## Conflicts of Interest

The authors declare no conflicts of interest.

## Data Availability

The data that support the findings of this study are available from the corresponding author upon reasonable request.
